# Ag@Nb_2_CT_*x*_ MXene composites for efficient photocatalytic degradation of norfloxacin and fleroxacin antibiotics

**DOI:** 10.1039/d5ra02680f

**Published:** 2025-07-18

**Authors:** Javeria Munir, Imran Haider Sajid, Syed Irfan, Syed Rizwan

**Affiliations:** a Physics Characterization and Simulations Lab (PCSL), Department of Physics and Astronomy, School of Natural Sciences (SNS), National University of Sciences and Technology (NUST) Islamabad 44000 Pakistan syedrizwan@sns.nust.edu.pk ihsajid@gmail.com; b State Key Laboratory of Environment-Friendly Energy Materials, Southwest University of Science and Technology Mianyang 621010 PR China

## Abstract

Two-dimensional (2D), MXenes' large surface area, great hydrophilicity, chemical stability, tunable electronic structure, and excellent electrical conductivity make them effective photocatalysts by enhancing light absorption and charge separation. To harness solar energy for photocatalysis, silver nanoparticles (Ag NPs), known for their catalytic properties, were incorporated into MXene sheets to synthesize Ag@Nb_2_CT_*x*_ composite by a cost-effective and straightforward electrostatic self-assembly method. The SEM images showed that Ag NPs were attached to the surface of 2D exfoliated MXene sheets. The photocatalytic efficiency of the Ag@Nb_2_CT_*x*_ composite was investigated for the photodegradation of Norfloxacin and Fleroxacin antibiotics, showing 74% and 68% degradation, respectively, in 120 minutes. The band gap of the prepared composite was tuned to 1.76 eV. Compared to Nb_2_CT_*x*_ MXene, Ag@Nb_2_CT_*x*_ composite exhibited good photodegradation due to its improved charge separation and less charge recombination rate. The photoluminescence spectra also showed that pristine MXene has the highest electron–hole pair recombination rate compared to all prepared Ag@Nb_2_CT_*x*_ composites. It is challenging to degrade antibiotics because of their strong chemical stability, so Ag@Nb_2_CT_*x*_ composite could be a potential candidate for commercial applications owing to its low-cost synthesis route.

## Abstract 2

Two-dimensional (2D) MXenes' large surface area, great hydrophilicity, chemical stability, tunable electronic structure, and excellent conductivity make them effective photocatalysts by enhancing light absorption and charge separation.

## Graphical abstract

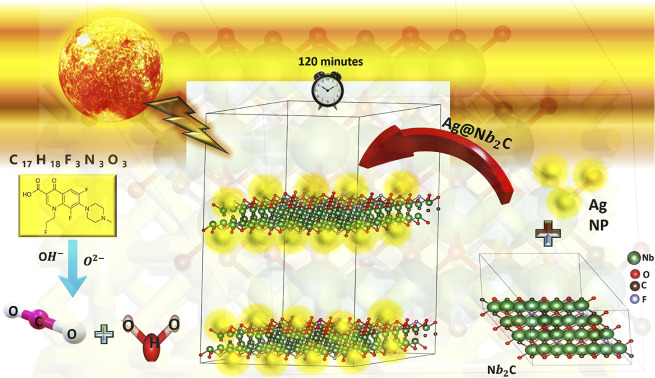

## 1. Introduction

Currently, among major environmental issues, water contamination due to organic pollutants has gained global attention.^1–3^ Specifically, organic pollutants, including dyes and antibiotics, cause water pollution. Dyes are discarded from textiles, food processing, cosmetics, and paper industries.^4–7^ Pharmaceutical waste like antibiotics and their residues are directly discharged into water bodies without prior treatment by several sources such as pharmaceutical industries, hospital sewage, human and livestock excretion, *etc.*^8,9^ Although these dyes and antibiotics are vital, their improper discharge in water reservoirs, even at low concentrations, can lead to serious issues for human health and the ecosystem because some dyes are very toxic and carcinogenic.^7,10^

Water purification is crucial to meet the need for potable water for the increasing population. Several advanced water purification techniques provide high-quality consumable water, including adsorption, biodegradation, reverse osmosis, and photocatalysis. Notably, photocatalysis is an effective method for dye, organic, and antibiotic degradation because of its advantages, such as its eco-friendliness, high efficiency, low-cost, and reusability.^4,11^ When the photocatalyst's surface is illuminated with light, electrons transfer from the valence band to the conduction band, leaving behind holes. These photo-induced electron–hole pairs react with water and form reactive oxygen species (ROS), leading to the degradation of organic pollutants. Several semiconductor-based photocatalysts, such as TiO_2_ and ZnO, have been discovered and reported for the degradation of organic pollutants. Still, their efficiency is not so good because of their wider band gaps.^11,12^

Among all the families of 2D nanomaterials, Gogotsi (2011) discovered a promising new 2D material known as MXenes. Due to their extraordinary properties, such as large surface area, great hydrophilicity, high electrical conductivity, fast ion transport mechanism, and large surface area, the MXene family has gained attention from researchers.^13–16^ MXenes (with basic formula M_*n*+1_X_*n*_T_*x*_) were selectively etched from MAX phases having basic formula M_*n*+1_AX_*n*_ where M is any transition metal (such as Nb, V, Ti, Mo *etc.*), A belongs to the Aluminum or Silicon family and X represents the Carbon, nitrogen or both where T_*x*_ in MXenes represents surface terminations or functional groups (such as O, F and OH) attached on MXene surface during etching process and *n* is an integer between 1 to 4. These surface terminations have a profound impact on MXene properties.^17^ In the MAX phase structure, M_*n*+1_X_*n*_ layers are sandwiched with pure A-element layers where M–A bonding is weaker than M–X bonding.^16,18,19^ Due to their great hydrophilic nature, chemical and thermal stability, large surface area, excellent mobility of electrons and holes and adjustable band gap, MXenes have become promising materials to be utilized as photocatalysts in environmental remediation strategies.^20^ However, the restacking of pristine MXene sheets may inhibit their practical applications as photocatalysts, but to prevent this restacking, the intercalation of nanoparticles acts as spacers between MXene sheets as well, and increased conductive channels is a good strategy to improve the photocatalytic efficiency.^11,21,22^ Recently, Nb_2_CT_*x*_ MXene has been recognized as a potential photocatalyst co-catalyst because it has a lower Fermi level than Ti_3_C_2_T_*x*_.^23–25^

In recent studies, nanostructured silver nanoparticle decoration has captured significant consideration for improved charge carrier separation, high electrical conductivity, high catalytic activity, its ability to enhance the visible light absorption attributed to surface plasmonic resonance (SPR) effect, and relatively low cost as compared to other metals.^26–28^ Silver nanoparticles embedded on MXene sheets exhibit good photocatalytic efficiency because of a suitable charge transfer mechanism.^29^ Pingtao *et al.*^30^ attained the highest photodegradation of methyl orange for Ag-doped ZnO/Graphene photocatalysts because of their effective charge separation. Owing to the spherical nano-sized Ag, we can achieve (a) an improved contact area of the photocatalyst and (b) enhanced charge transfer due to high electrical conductivity. The uniform distribution of Ag NPs on MXene sheets can enhance the conductive channels for better charge transportation between MXene layers.^31^

In this work, we report the synthesis of pristine MXene and various Ag@Nb_2_CT_*x*_ nanocomposites as photocatalysts by the self-assembly of Ag NPs on Nb_2_CT_*x*_ MXene nanosheets through van der Waals interactions. Ali *et al.* and Haider *et al.*^21,32^ have also observed these interactions between Ag NPs and MXene. The structural, morphological, and optical stability of as-prepared nanocomposites is also reported in this study by using different characterization techniques. Among these nanocomposites, C_2_ (MXene : Ag NPs: 1 : 0.5) showed good photocatalytic efficiency compared to pristine MXene and other nanocomposites as photocatalysts. The author firmly believes that the study of incorporation of Ag NPs in Nb_2_CT_*x*_ MXene in this research could pave more opportunities to examine and explore the properties of other different MXenes that have not been widely studied yet, besides Ti_3_C_2_T_*x*_.

## 2. Experimental section

**Fig. 1:**
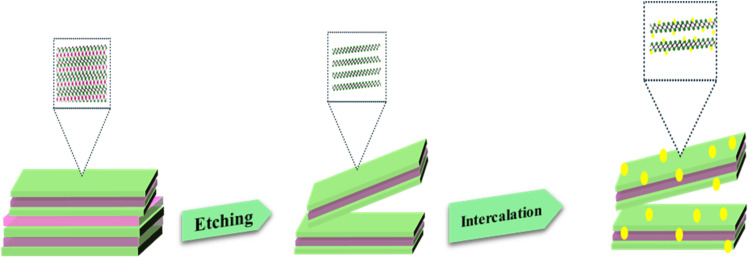
Schematic interpretation for synthesis of Nb_2_CT_*x*_ MXene and Ag@Nb_2_C composite.

The method used to synthesize the Nb_2_CT_*x*_ MXene and Ag@Nb_2_CT_*x*_ composites is depicted schematically in Fig. 1.

### 2.1. Materials and reagents

The MAX powder (Nb_2_AlC, purity 99%) was purchased from Carbon Ukraine company. Silver Nitrate (AgNO_3_, ACS reagent, 99%), hydrofluoric acid (48% purity), deionized water (DI, purity 99%), cetyltrimethylammonium bromide (CTAB, purity 99%) were purchased from Sigma Aldrich and Sodium borohydride (NaBH_4_, CAS No. 16940-66-2) was purchased from Dae-Jung Chemicals and Materials Co. Ltd Korea. All reagents and chemicals were utilized in their analytical grade without any purification.

### 2.2. Synthesis of Nb_2_CT_*x*_ MXene

The Nb_2_CT_*x*_ MXene was prepared using a wet chemical etching method from the Nb_2_AlC MAX phase. 1 g milled powder of MAX (Nb_2_AlC) phase was gradually immersed in 20 ml HF (47%) aqueous solution and stirred using a Teflon-coated magnetic stirrer at 300 rpm for 96 hours at 55 °C in a Teflon beaker. The etched powder was then washed several times with deionized water until the pH of the supernatant exceeded 6. The solution was subsequently filtered by filtration assembly. The dried powder on filter paper was placed in a vacuum oven at 60 °C overnight. Finally, the dried powder was collected and kept in an Eppendorf tube in a vacuum desiccator.

### 2.3. Synthesis of silver nanoparticles

The homogenous solution of silver nanoparticles was synthesized through the chemical reduction method of AgNO_3_ salt. In brief, 50 ml of 9.0 mM aqueous solution containing silver nitrate AgNO_3_ and 0.1 wt% cetyltrimethylammonium hydroxide (acts as a surfactant and gives a positive charge to the silver) was placed for constant stirring at room temperature. The stirring was continued for the next 30 minutes to achieve homogeneity. After that, the 10 ml of 8 mM refrigerated cold sodium borohydride (3.1 mg) was added dropwise into the prepared mixture solution. Immediately, the solution turned bright yellow, indicating the formation of silver nanoparticles (Ag NPs). The solution was kept under stirring for 1 hour.

### 2.4. Synthesis of Nb_2_CT_*x*_ and Ag@ Nb_2_CT_*x*_ composite

In synthesizing the Nb_2_CT_*x*_/Ag NPs composite, the electrostatic self-assembly method was used to load Ag NPs on Nb_2_CT_*x*_ MXene sheets. An aqueous 100 mg Nb_2_CT_*x*_ MXene solution was prepared in 100 ml DI and stirred at 40 °C for 1 hour. Then, 50 ml 9 mM Ag NPs colloidal solution was added dropwise in the MXene. The solution was left at continuous stirring for 30 minutes to get a homogeneous mixture. Then, the mixture was thoroughly washed with DI and filtered. The filtrate was dried overnight in a vacuum oven at 60 °C to obtain the final product. By following a similar fashion, three solution volume ratios 1 : 0.25, 1 : 0.5 and 1 : 0.75, of MXene : Ag NPs were synthesized to prepare the samples containing 0.25%, 0.5% and 0.75% weight ratios of Ag NPs to MXene nanosheets by preparing 50 ml of 3 mM, 6 mM and 9 mM Ag NPs colloidal solution and named them C_1_ (1 : 0.25), C_2_ (1 : 0.5) and C_3_ (1 : 0.75) respectively.

### 2.5. Photocatalytic degradation measurements

The photocatalytic performance of as-prepared materials Nb_2_CT_*x*_ and Ag@Nb_2_CT_*x*_ were investigated against two antibiotics, norfloxacin and fleroxacin, for their photodegradation in a glass vessel using a 400 W Halogen lamp as a light source to initiate the photocatalytic process. 15 mg of photocatalyst was added in 30 ml antibiotic-contaminated (10 mgL^−1^) solution. The dispersion was stirred in the dark for 20 minutes to attain the adsorption–desorption equilibrium before visible light exposure. Samples of 2 ml were taken at regular 30-minute intervals to measure antibiotic degradation. The samples were separated by centrifugation. Then, the supernatant is analyzed by measuring the maximum absorbance using a double-beam UV-vis spectrophotometer. The same procedure was conducted by varying the concentrations of the photocatalyst, *i.e.*, 3 mg and 30 mg, in the antibiotic solution. The photodegradation efficiency was calculated using the formula.

1 Degradation (%) = *C*_o_ − *C*_*t*_/*C*_o_ × 100

where *C*_o_ is the initial concentration of contaminated solution and *C*_*t*_ is the concentration of contaminated solution at any time instant *t*.^33–36^

### 2.6. Material characterizations

The structural properties of the prepared materials were analyzed through X-ray diffraction (XRD) with wavelength 1.54 Å from a monochromatic Cu-Kα source (Burk, D8Advance). To analyze the chemical structures and bonding, Raman Spectroscopy (532 TEC-Ci) was used, whereas to investigate the band gaps and percentage degradation further, Ultraviolet-visible (UV-vis) Spectroscopy (Lambda 365PekinElmer) was utilized. The scanning electron microscope (SEM) was used to examine the surface morphology of the prepared materials through a Hitachi s-4800FEG. The energy dispersive X-ray spectroscopy (EDX) explored the elemental composition. The Fourier Transform Infrared spectroscopy was used to investigate the chemical composition and surface terminations attached to the materials. The recombination rate of charge carriers in materials was analyzed using photoluminescence (PL) spectra.

## 3. Results and discussions

**Fig. 2:**
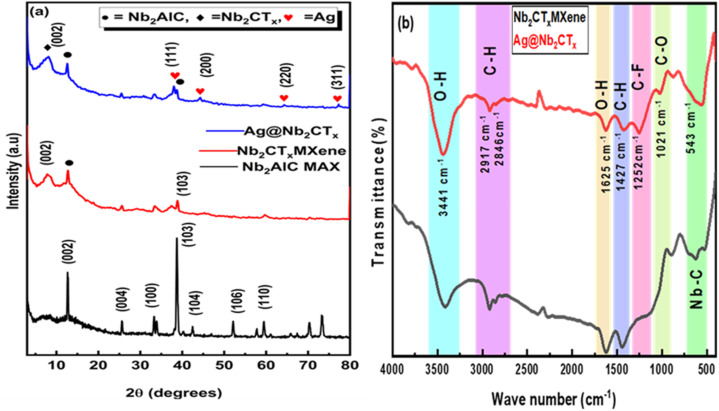
(a) XRD spectra of Nb_2_AlC MAX (black), Nb_2_CT_*x*_ MXene (red), Ag@Nb_2_CT_*x*_ (C_2_) (blue), (b) FTIR spectra of Nb_2_CT_*x*_ MXene, and Ag@Nb_2_CT_*x*_ (C_2_). The figure should be in high magnification.

XRD provides detailed information about the structure of the material. Fig. 2(a) compares the parent Nb_2_AlC MAX phase, exfoliated Nb_2_CT_*x*_ MXene, and Ag@Nb_2_CT_*x*_ composite. The sharp and intense peaks in the Nb_2_AlC MAX phase are evident in its crystalline structure. The Nb_2_AlC MAX phase depicts its major peaks at 2*θ*: 12.65^°^, 25.7^°^, 33.3^°^, 38.7^°^, 42.7^°^, 52.1^°^, 59.5^°^ corresponding to planes (002), (004), (100), (103), (104), (106) and (110) respectively and is compatible to previous reported data (JCPDSNo:00-030-0033).^37,38^ Meanwhile, the all-other peaks are intermetallic.^39^ The (002) peak at 12.65^°^ is shifted at the lower angle in the case of MXene at 7.9^°^ after HF treatment, so its *d*-spacing increases from 6.99 Å to 11.18 Å and c-lattice parameter increases from 13.98 Å to 22.36 Å, which shows the removal of Al layer from the MAX phase.^40^ The ions/water pillars or etching products are intercalated among MXene layers, causing the shifting of the (002) peak in the case of MXene from MAX.^41,42^ The un-etched MAX is also present, as seen from a tiny (103) peak at 38.7°, which is Aluminum. The broadening and shifting of the (002) peak from 7.9° to 8.2° shows the presence of Ag nanoparticles between MXene layers.^43,44^ The corresponding *d*-spacing also decreases from 11.04 Å to 10.51 Å, and c-lattice parameters decrease from 22.08 Å to 21.03 Å. Some extra minor peaks (111), (200), (220), and (311) corresponding to 2*θ*: 38.1^°^, 44.3^°^, 64.5^°^, and 77.4^°^ have emerged due to Ag-NPs in Ag@Nb_2_CT_*x*_ composite and are in good agreement with Ag NPs JCPDS card no. 04-0783.^45^ Our synthesis method, based on electrostatic self-assembly, differs significantly from high temperature aqueous processing or hydrothermal routes known to oxidize Nb_2_CT_*x*_ into Nb_2_O_5_ (acts as good semiconducting photocatalyst) or rutile/anatase phase TIO_2_ phase in Ti_3_C_2_ MXene, previously reported by Peng *et al.*, Xu *et al.* and Peng *et al.*^46–48^ This technique avoids high temperature, pressure, and prolonged exposure to oxidizing environments. As a result, no oxidation to Nb_2_O_5_ occurred, which is clearly supported by the absence of Nb_2_O_5_ peaks in the XRD patterns at 2*θ* = 26.62° and 45.98° in our reported work but Peng *et al.* reported the formation of Nb_2_O_5_ nanorods in his report due to hydrothermal synthesis route.^46^ The photocatalytic activity observed is therefore attributed to the pristine or surface-functionalized Nb_2_CT_*x*_ MXene, not any Nb_2_O_5_ phase. The FTIR spectra of pristine Nb_2_CT_*x*_ MXene and Ag@Nb_2_CT_*x*_ composite were recorded in the wavenumber range of 400 cm^−1^ to 4000 cm^−1^, as shown in Fig. 2(b).^49,50^ The spectra can be divided into the functional group region (4000 cm^−1^ to 1000 cm^−1^) and the fingerprint region (below 1000 cm^−1^). The peak at 3441 cm^−1^ is attributed to O–H bond stretching vibrations, while the peaks at 2917 cm^−1^ and 2846 cm^−1^ correspond to C–H bonds stretching vibrations. The peaks observed at 1625 cm^−1^, 1427 cm^−1^, and 1021 cm^−1^ are associated with O–H bending vibrations, C–H bonds bending vibrations and C–O bonds stretching vibrations, respectively.^50,51^ A C–F band at 1252 cm^−1^ suggests asymmetric functionalization of the MXene sheets. The Ag@Nb_2_CT_*x*_ composite shows a higher density of surface functional groups than pristine MXene, as confirmed by similar findings in the Raman data. The peak at 543 cm^−1^ in the fingerprint region corresponds to the Nb–C mode of vibrations in MXene, and a similar but more intense peak in the composite material due to rich surface terminations or functional groups attached on the composite during synthesis process further indicates the successful synthesis of the Ag@Nb_2_CT_*x*_ composite.^49,52^

**Fig. 3:**
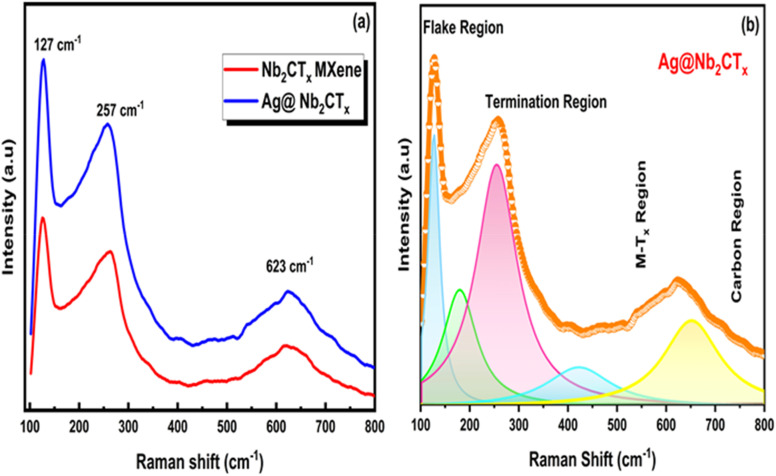
(a) Raman spectra of Nb_2_CT_*x*_ MXene and Ag@Nb_2_CT_*x*_ (C_2_), (b) deconvoluted Raman spectra of Ag@Nb_2_CT_*x*_ (C_2_).

Raman spectroscopy is a non-destructive analytical method that provides information regarding chemical structure, phase, crystallinity and molecular interactions. Raman spectrum was observed for the exfoliated MXene and Ag@Nb_2_CT_*x*_ composite within the range of wave numbers of 100 to 800 cm^−1^, as shown in Fig. 3(a). For Nb_2_CT_*x*_ MXene, the characteristic peaks appear at 127 cm^−1^, 257 cm^−1^, and 410 cm^−1^, representing the in-plane *E*_g_ vibrations of Nb atoms and their associated surface groups and are matched with the previous data.^37,53^ The peak at 623 cm^−1^, corresponding to the A_1g_ modes of MXene, further confirms these features. When Nb_2_CT_*x*_ MXene is combined with Ag, no significant peak shifts are observed. This suggests that the Nb_2_CT_*x*_ MXene retains its high quality after composite formation, with Ag particles responsible for the enhanced peak intensity. Notably, since Ag itself does not produce peaks in Raman spectroscopy, the enhancement is attributed to Ag particles within the Nb_2_CT_*x*_ structure. Fig. 3(b) illustrates the deconvoluted Raman spectra of the Ag@Nb_2_CT_*x*_ composite, where the Lorentz function had been used for the deconvoluted and fitted graph. The prominent peak at 127 cm^−1^ belongs to the flake region and shows Nb–C vibrations. The in-plane symmetric vibrations near 260 cm^−1^ are identified as A_1g_ out-of-plane vibrations of the metal atoms. The region from 200 cm^−1^ to 500 cm^−1^ is associated with the outer layer of metal and carbon atoms and the mixed functional groups. The region of 550 cm^−1^ to 800 cm^−1^ results from multiple in and out-of-plane carbon vibrations, with vibrations between 600 cm^−1^ and 800 cm^−1^ being denoted as asymmetric, appearing only in terminated MXenes.^49,54–56^

**Fig. 4:**
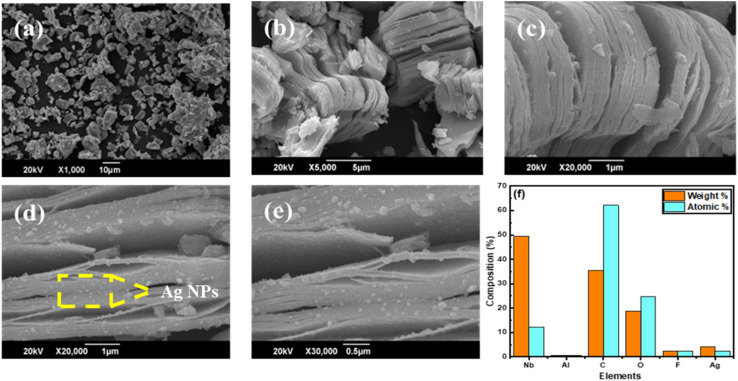
SEM images of (a) Nb_2_AlC MAX, (b and c) pristine Nb_2_CT_*x*_ MXene, (d and e) Ag@Nb_2_CT_*x*_ (C_2_), (f) EDX plot for the elemental analysis of Ag@Nb_2_CT_*x*_.

Scanning electron microscopes play a crucial and beneficial role in assessing defects and exploring diverse materials' topography, composition, and surface features. Fig. 4(a) displays the SEM image of the Nb_2_AlC MAX phase, highlighting its lump-like morphology. This image provides insight into the surface topography and texture of the MAX phases and reveals the grain size. After etching Nb_2_AlC, Fig. 4(b and c) depicts the layered structure of exfoliated MXene. Additionally, surface impurities can be observed on the MXene sheets, indicating the presence of aluminum, fluoride, and unreacted MAX phase. Some impurities also arise from the functional groups that attach to the MXene surface after etching. The SEM images of the Ag@Nb_2_CT_*x*_ composite, which represents the successful intercalation of Ag nanoparticles on MXene surface and sheets, are shown in Fig. 4(d and e). Fig. 4(f) shows the EDX plot of the Ag@Nb_2_CT_*x*_ composite, which shows the presence of all elements in the composite like Nb, C, F, Al, and Ag. A peak of fluorine and oxygen are attached as terminations to the MXene sheets as a consequence of the HF etching and washing process.^57^ There is also a peak of Ag, which shows the successful decoration of silver nanoparticles on the MXene surface and sheets. The tiny peak of Al shows minor traces of unreacted MAX phase in the EDX plot.

**Fig. 5:**
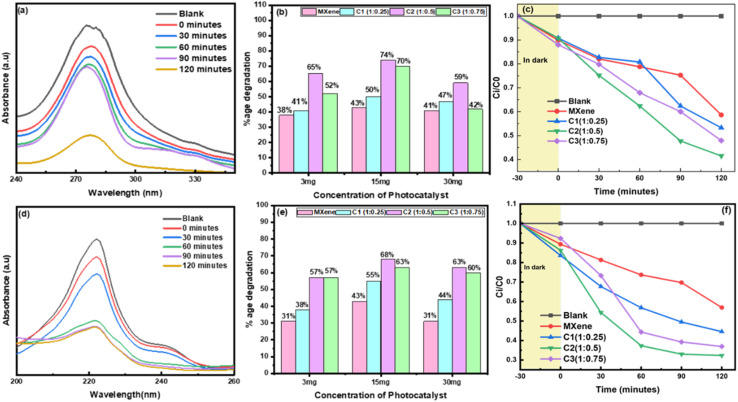
(a and d) Absorbance spectra of Ag@Nb_2_CT_*x*_ (C_2_) showing the degradation efficiency of norfloxacin and fleroxacin antibiotics from solutions at different times respectively, (b and e) comparison of different photocatalysts at various ratios, (c and f) photocatalytic degradation of norfloxacin and fleroxacin antibiotics in the presence of pristine MXene and nanocomposites.

The photocatalytic degradation of two antibiotics, norfloxacin and fleroxacin, was examined to investigate the photocatalytic performance of Nb_2_CT_*x*_ MXene and the Ag@Nb_2_CT_*x*_ composite. The blank experiment without any photocatalyst indicates that norfloxacin and fleroxacin were hardly degraded, reflecting that the direct minor photolysis of norfloxacin and fleroxacin after visible light exposure can be neglected. The photodegradation efficiencies of norfloxacin and fleroxacin are 43% and 42% in the presence of MXene for 120 minutes, respectively. Of course, photocatalytic efficiency would increase after the Ag NPs were introduced. Both norfloxacin and fleroxacin showed 13% photodegradation for Ag@Nb_2_CT_*x*_ composite in the dark due to adsorption. But when exposed to light, the photocatalytic efficiency of Ag@Nb_2_CT_*x*_ was increased with the increase of the content of Ag NPs, and the material C_2_ (MXene : Ag: 1 : 0.5) showed the highest photodegradation efficiency with 74% and 68% for norfloxacin and fleroxacin, respectively. The absorption spectra of the Ag@Nb_2_CT_*x*_ composite for norfloxacin and fleroxacin are recorded and illustrated in Fig. 5(a and d).

As the content of Ag NPs increases, the Ag@Nb_2_CT_*x*_ forms a large interface contact, which improves the separation and migration of photo-generated charge carriers from Nb_2_CT_*x*_ to Ag NPs, leading to improved and high photo-degradation efficiency. Although incorporating Ag NPs to Nb_2_CT_*x*_ MXene promotes the charge transfer from Ag NPs to Nb_2_CT_*x*_ MXene, photodegradation efficiency decreases when the Ag NPs content is more than 0.5% weight ratio to MXene. This result shows that the optimization of incorporated material plays a crucial role.^23^ The optimized ratio of Ag NPs in Nb_2_CT_*x*_ MXene could boost the photodegradation efficiency of the Ag@Nb_2_CT_*x*_ composite material. Similarly, contrary to the optimized ratio, optimization of the concentration of photocatalyst is also essential. Norfloxacin and fleroxacin showed 65% and 57% photo-degradation with 3 mg/30 ml, 74% and 68% photo-degradation with 15 mg/30 ml, and 59% and 63% photo-degradation with 30 mg/30 ml for Ag@Nb_2_CT_*x*_ composite material. The comparison of all the 4 materials pristine Nb_2_CT_*x*_ MXene and Ag@Nb_2_CT_*x*_ (C_1_ (1 : 0.25), C_2_ (1 : 0.5) and C_3_ (1 : 0.75)) as photo-catalysts with different concentrations is shown in Fig. 5(b and e). This can be interpreted because while increasing the photocatalyst concentration distributes more active sites, the powder particles may accumulate at high concentrations and decrease efficiency.^58^ So, 15 mg/30 ml (or 500 mg L^−1^) concentration of Ag@Nb_2_CT_*x*_ was opted as the optimized concentration for norfloxacin and fleroxacin antibiotic removal.^59^ From Fig. 5(c and f), the black line represents the negligible photocatalytic degradation of norfloxacin and fleroxacin without any photocatalyst. MXene shows average photo-degradation, and the C_2_ (1 : 0.5) material shows good photocatalytic activity, while the other two materials, C_1_ (1 : 0.25) and C_3_ (1 : 0.75)) also showed appreciable removal activity for norfloxacin and fleroxacin antibiotics. Ag nanoparticles bind to Nb_2_CT_*x*_ MXene sheets and surface and might form a network or act as bridge that enhance conductive channels between Ag and MXene layers for efficient charge transport.^32^ This fast and smooth charge transfer prevents recombination of electrons and holes, thus boosting the photocatalytic performance when these separated electrons and holes participate in redox reactions.

The UV-vis spectrophotometer obtained the absorption spectra of pristine Nb_2_CT_*x*_ MXene and Ag@Nb_2_CT_*x*_ composite. A well-known Kubelka–Munk technique was used to estimate the absorption spectra and relation for photon energy *hν* for the allowed transition and absorption coefficient *α* is

2 (*αhν*) = *A*(*hν* − *E*_g_)^*n*/2^

**Fig. 6:**
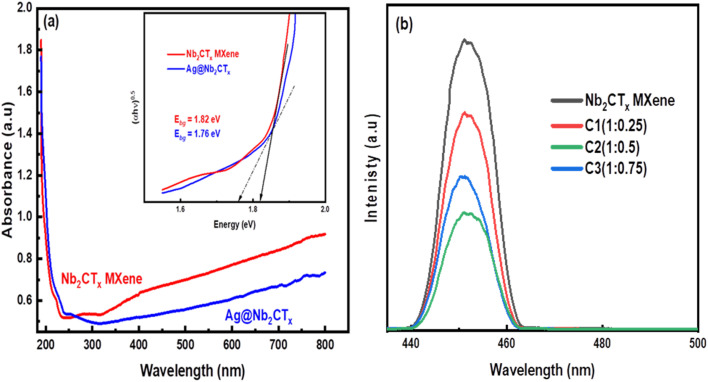
(a) Absorbance and band gap of pristine Nb_2_CT_*x*_ MXene and Ag@Nb_2_CT_*x*_ (C_2_), (b) Photoluminescence (PL) spectra of pristine MXene and nanocomposites.

In eqn (2), *A* is a constant, *ν* is the frequency of light, *h* is Planck's constant, *α* is the absorbance of light, *E*_g_ is band gap energy, and *n* is a positive integer.^60^ The absorption spectra of both materials were measured at room temperature. To calculate the band gap of pristine MXene and Ag@Nb_2_CT_*x*_ materials, the Tauc plot method was used, as shown in the inset graph in Fig. 6(a). The calculated band gap for the pristine MXene and Ag@Nb_2_CT_*x*_ was 1.82 eV and 1.76 eV, respectively, which demonstrates that Ag@Nb_2_CT_*x*_ is preferable and suitable for photocatalytic application than pristine Nb_2_CT_*x*_ MXene. Although Ag NPs are adsorbed onto the MXene surface while MXene retains its overall atomic structure, but their presence might still affect the overall electronic environment. As reported by Haider *et al.* these NPs bind on MXene surface and forms conducting channels, improving movement and separation of charge carriers and might introduce new electronic states at surface and interface.^32^ It might lead to slight modifications in overall electronic structure which might cause the apparent band gap to decrease slightly. When Ag@Nb_2_CT_*x*_ is exposed to light, semiconducting Nb_2_CT_*x*_ MXene generates photo generate electron hole pairs, electrons move on the Ag NPs from conduction band of MXene while holes remain in the valence band of MXene. These electrons on Ag NPs and hole participate in redox reactions to degrade pollutants in water.^34,46,61,62^Fig. 6(b) presents the PL spectra of pristine Nb_2_CT_*x*_ MXene and Ag@Nb_2_CT_*x*_ composites at various ratios. The pure Nb_2_CT_*x*_ sample exhibits a primary emission peak around 451 nm.

The spectra intensity represents the charge carrier's recombination rate. Compared to pristine Nb_2_CT_*x*_ MXene, the PL intensity of the Ag@Nb_2_CT_*x*_ composites decreases significantly at the same wavelength, indicating a lower charge recombination rate in the Ag@Nb_2_CT_*x*_ composites. Generally, a lower charge recombination rate corresponds to a reduced PL intensity, enhancing photocatalytic activity. In Fig. 6(b), the black line represents the PL spectrum of the pristine Nb_2_CT_*x*_ MXene. The higher PL intensity for this sample suggests a high charge recombination rate, which correlates with lower photocatalytic activity. Conversely, the green curve shows the lowest intensity values, corresponding to the lowest charge recombination rate. This reduced recombination rate enhances the photocatalytic activity, with the green curve representing the Ag@Nb_2_CT_*x*_ C_2_ (1 : 0.5) composite. This sample demonstrates the highest photocatalytic efficiency, achieving 74% and 68% photo-degradation for norfloxacin and fleroxacin antibiotics. This is because of the porous structure of exfoliated 2D MXene sheets which trap electrons, improve the electron–hole separation, and boost photocatalytic efficiency.

Also, the lower charge recombination rate of all composites compared to pristine MXene leads to enhanced utilization of active sites on MXene sheets, thereby improving photocatalytic performance. As the light falls on composites, the radicals are generated, showing nanoparticle activation. At first, the photo-generated electron–hole pairs are formed due to the transfer of photo-excited electrons from the VB to the CB. Then, these excited electrons and holes participate in redox reactions to degrade the harmful organic pollutants, dyes, and antibiotics into less harmful, simpler molecules.

**Fig. 7:**
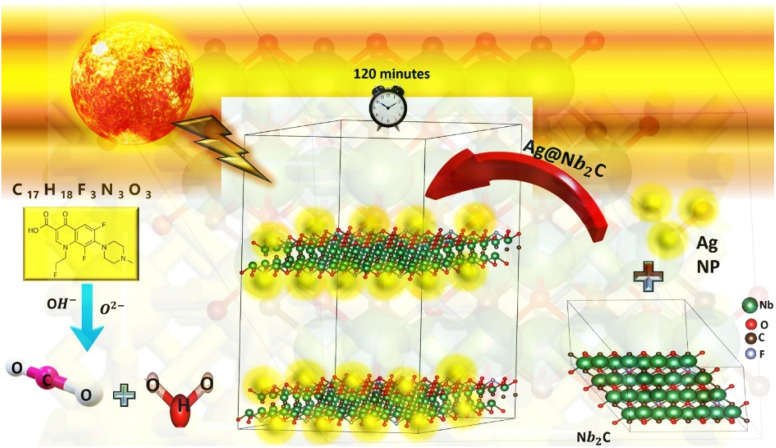
Ag@Nb_2_C composite antibiotic degradation mechanism.

The proposed mechanism of photocatalysis depends on the oxidation–reduction reactions; on the surface of photocatalyst, in the presence of electrons and holes, is shown in Fig. 7. Pristine MXene act as metallic or metal phase co-catalyst but the terminated MXene or MXene based composites behave as semiconductors due to abundance of active sites and porous structure during the synthesis process.^63–65^ In the Ag@Nb_2_CT_*x*_ material, Nb_2_CT_*x*_ MXene act as semiconductor active sites due to surface terminations (such as –F, –O, –OH *etc.*) which induce band gap and allow visible light absorption and Ag act as reduction co catalyst.^46^ When expose to light, Nb_2_CT_*x*_ MXene generates photogenerated electron–hole pairs. These photogenerated electrons then transfer on the surface adsorbed Ag NPs which act as electron sinks and redox active sites, help to reduce the absorbed oxygen to form superoxide radical (˙O_2_^−^). While holes remain on the MXene surface and oxidize water or hydroxide ions into hydroxyl radicals (˙OH). These radicals are highly reactive species and break antibiotics molecules from water into harmless and simple end products like H_2_O and CO_2_. The abundant surface terminations provide active sites for both pollutant adsorption and ROS formation. Additionally, Ag nanoparticles enhance visible-light utilization through localized surface plasmon resonance (LSPR) and help suppress electron–hole recombination.^46^ Together, this synergy between semiconducting Nb_2_CT_*x*_ and plasmonic Ag drives efficient photocatalytic degradation of organic pollutants under visible light.

These end products can be converted into hydrocarbons like methane, *etc.*, by further reduction. Electron–hole pairs are generated when light falls on the photocatalyst surface (eqn (3)). Electrons transfer on Ag while exfoliated MXene sheets behave as hole acceptors due to negatively attached surface terminations and functional groups and trap holes to increase the separation of charge carriers and help to reduce recombination rate of electron hole pairs (eqn (4) and (5)). Electrons reduce absorbed oxygen and O_2_ in water into superoxide radicals while holes oxidize the water molecules into hydroxyl radical ions,˙OH^−^ (eqn (6) and (7)). These ROS react with antibiotics and degrade them to give simple end products like CO_2_ and H_2_O.^66–68^

The proposed photodegradation procedure is demonstrated by the following equations^66,68^

3 Ag@Nb_2_CT_*x*_ MXene + *hν* → e^−^ + h^+^

4 e^−^ + (Ag@Nb_2_CT_*x*_ MXene) → e^−^(Ag trapped electrons)

5 h^+^ + (Ag@Nb_2_CT_*x*_ MXene) → (MXene trapped h^+^)

6 e^−^ +O_2_ → ˙O_2_^−^

7 h^+^ (trapped in MXene) + H_2_O → ˙OH^−^

8 ˙OH^−^ + Antibiotic → CO_2_ +H_2_O

9 ˙O_2_^−^ + Antibiotic → CO_2_ +H_2_O

Norfloxacin antibiotic has molecular formula C_16_H_18_FN_3_O_3_ and when ROS species degrade antibiotics then we get less toxic degradation products such as H_2_O and CO_2_.^69^ It can be seen that C_2_ is a promising photocatalyst. This shows good photodegradation because of less electron–hole recombination rate and narrow band gap (1.76 ev) compared to other nanocomposites. The low band gap is due to the doping of Ag NPs that further generate more ROS species by oxidation–reduction reactions. Hence, the production of these ROS species allows for the photodegradation of contaminants and organic pollutants in water.^34,68^ The Ag@Nb_2_CT_*x*_ photocatalyst achieve 74%and 68% degradation of norfloxacin and fleroxacin within 120 minutes under visible light irradiation. Although this degradation efficiency is moderate as compared to other materials discussed in Table 1, the synthesis of the composite is straightforward, simple and cost effective. The catalyst operates effectively under relatively low catalyst dosage and ambient conditions, making it promising for practical applications where energy efficiency and ease of preparation are important. Additionally, the incorporation of Ag nanoparticles enhances the charge separation efficiency in MXene, contributing to the observed photocatalytic activity. Furthermore, no MXene based photocatalysts have been reported till date for fleroxacin.

**Table 1:** Comparison of MXene based and silver based photocatalysts for norfloxacin and fleroxacin antibiotics

Sr. no.	Catalyst	Organic pollutant with conc. (ppm)	Catalyst conc. (mg mL^−1^)	% Age degradation	Light source	Time (minutes)	Ref.
1	ZnO–Ti_3_C_2_T_*x*_ composites	NOR 20	100 mg/50 ml	90	300 W xenon lamp	240	70
2	C–TiO_2_	NOR 10	10 mg/25 ml	74	500 W halogen lamp	150	71
3	NiFe-LDH/MXene	NOR 20	20 mg/50 ml	98	300 W xenon lamp	240	72
4	Ag@Nb_2_CT_*x*_	NOR 10	15 mg/30 ml	74	400 W halogen lamp	120	
5	g-C_3_N_4_/PPy/Ag	FLE 20	50 mg/50 ml	90.2	250 W xenon lamp	180	73
6	Ag@Nb_2_CT_*x*_	FLE 10	15 mg/30 ml	68	400 W halogen lamp	120	

## 4. Conclusion

Ag nanoparticles were synthesized using the chemical reduction method. The 2D MXene sheets were synthesized from their parent MAX by wet chemical etching of aluminum using HF as an etchant. The Ag@Nb_2_CT_*x*_ nanocomposites were prepared using the electrostatic self-assembly method with various doping concentrations. The XRD and SEM results also indicated the successful formation of nanocomposites. The nanocomposites showed a high electron–hole pair generation rate and low recombination rate as compared to pristine MXene. The photodegradation of norfloxacin and fleroxacin antibiotics was higher in the case of C_2,_ showing 74% and 68%, respectively, in 120 minutes compared to pristine MXene and other nanocomposites. Although it's challenging to degrade antibiotics, a highly efficient Ag@Nb_2_CT_*x*_ nanocomposite made it possible without any co-catalyst. The nanocomposites synthesized and reported as photocatalysts are the novel and best candidates for photocatalytic activity and are unique, making them promising candidates for commercial applications owing to their cost-effective route.

## Conflicts of interest

There are no conflicts to declare.

## Data Availability

Data will be made available on request.
